# Age dependent association of endometrial polyps with increased risk of cancer involvement

**DOI:** 10.1186/1477-7819-3-8

**Published:** 2005-02-09

**Authors:** Denise Hileeto, Oluwole Fadare, Maritza Martel, Wenxin Zheng

**Affiliations:** 1 Department of Pathology, Yale University School of Medicine, New Haven, CT, 06520-8070 USA; 2 Department of Obstetrics and Gynecology, Yale University School of Medicine, New Haven, CT, 06520-8070, USA

## Abstract

**Background:**

Endometrial polyps (EMPs) are commonly encountered in routine surgical pathology practice, but opinions differ on whether they are intrinsically a marker for concurrent or subsequent malignancy. The objectives of the present study are 1) to investigate the age-group in which EMP are most commonly encountered 2) to document the age-group in which EMP are most commonly associated with malignancies 3) To investigate whether the age of diagnosis of the various carcinoma subtypes in EMPs is congruent with published data on similar malignancies arising in non-polypoid endometrium and 4) To investigate whether the histologic subtype distribution of malignancies associated with EMPs are similar or different from the distribution of malignancies arising from non-polypoid endometrium based on published data.

**Patients and methods:**

All cases of EMPs were retrieved from the files of Yale-New Haven Hospital for the period 1986–1995. The patients were divided into 5 age groups: Each group was further subclassified based on an association (or lack thereof) of EMPs with endometrial carcinoma. Chi-square test was used to compare the proportion of malignancy associated EMPs between the age groups.

**Results:**

We identified 513 EMPs, of which 209 (41%) were from biopsy specimens and 304 (59%) from hysterectomy specimens. Sixty six (13%) of all EMPs were malignant. The 66 malignant EMPs included 58 endometrioid, 6 serous, 1 carcinosarcoma, and 1 clear cell carcinoma. In age group >35, only 1(2.5%) of 40 EMPs was associated with endometrial malignancy. In contrast, 37(32%) of 115 EMPs were associated with malignancy in the age group > 65. The frequency of malignant EMPs increased with age and reached statistical significance in the age group >65 (p < 0.001). The most common histologic type of malignancy was endometrioid adenocarcinoma.

**Conclusions:**

EMPs show statistically significant age dependent association with malignant tumor involvement. Careful search for malignancy, particularly in women with multiple risk factors is advised in daily practice. Additional studies are needed to address the histological features and immunohistochemical profiles in the context of association between endometrioid and high-grade endometrial carcinoma and endometrial polyps.

## Background

Endometrial polyps (EMPs) are generally considered benign proliferative lesions and are commonly encountered in routine surgical pathology practice. The usual histological pattern of endometrial polyps is characterized by irregular proliferative glands, with a fibrotic stroma containing thick-walled blood vessels [1]. The morphologic diversity of endometrial polyps is reflective of the morphologic spectrum of the background endometrium from which EMPs arise. As such, EMPs may range from atrophic to hyperplastic to carcinomatous. However, opinions differ on whether EMPs are intrinsically a marker for concurrent or subsequent malignancy. Endometrial polyps were identified in 12–34% of uteri containing endometrial carcinoma in two earlier studies [2,3]. In another case-control study examining previous pathology in women diagnosed with endometrial carcinoma, endometrial polyps were twice as likely to be detected than in the control group [4]. Rarely, serous endometrial intraepithelial carcinoma (EIC), the presumptive early form of uterine papillary serous carcinomas, may be identified as very minute foci in EMPs [5,6]. This finding may be interpreted as the EMP homologue of similar changes that are occasionally identified in non-polypoid atrophic endometrium. However, given that nonrandom chromosomal aberrations and monoclonality that have been demonstrated in EMPs [7,8], an alternate interpretation is that molecular and/or cytogenetic alterations inherent to EMPs facilitate a neoplastic transformation. The latter interpretation would imply that endometrial polyps are a risk factor for the development of endometrial tumors. And indeed a possible association between endometrial polyps and endometrial malignancy in postmenopausal women has been suggested couple of decades ago [4]. However, there is no direct evidence for a greater propensity of polypoid endometrium to undergo malignant change as compared to the adjacent normal endometrium, and EMP may simply represent am embodiment of the greater propensity of the host endometrium to develop proliferative/neoplastic changes in general [9]. A recent study designed and conducted to investigate the pathological significance of EMPs and their association with pre-malignant and malignant conditions failed to supply evidence of such association. That study involved a large cohort of patients seen in outpatient hysteroscopy clinic for abnormal uterine bleeding. To determine the magnitude of malignant potential among polyps, the authors compared the pathological findings in polyps with non-polypoid specimens. The comparative analysis established that endometrial hyperplasia was more frequent in endometrial specimens with polyps, but the incidence of frank carcinoma in polypoid and non-polypoid endometrium remained the same. Although not age stratified, the study showed that in abnormal uterine bleeding, hyperplasia presented more frequently in women with EMPs compared to those without polyps, but cancer involvement regardless of the histological pattern was not significantly different [9]. Similar results and failure to establish any association of endometrial polyps and carcinoma were demonstrated in another recent study dealing with endometrial polyp characteristics in menopausal women on hormonal replacement therapy [10].

Most standard pathology texts list endometrial polyps as being most prevalent in perimenopausal women and suggest possible association between polyps and malignant involvement [9,11]. However, there has been no detailed age-based analysis of the incidence and malignant involvement of EMPs. In this report, age-related differences in the incidence of EMP at the time of diagnosis in the practice of a busy academic center is examined, with a detailed analysis of the incidence and histologic subtypes of malignancies associated with EMPs. The objectives of the study are 1) to investigate the age-group in which EMP are most commonly encountered in routine surgical pathology practice 2) to document the age-group in which EMPs are most commonly associated with malignancies 3) To investigate whether the age of incidence of the various carcinoma subtypes in EMPs at the time of diagnosis is congruent with published data on similar malignancies arising in non-polypoid endometrim and 4) To investigate whether the histologic subtype distribution of malignancies associated with EMPs is similar or significantly different from the distribution of malignancies arising from non-polypoid endometrium.

## Patients and methods

### Case retrieval and pathologic classifications

All cases with a diagnosis of EMP were retrieved from the computerized database of the Pathology Department at Yale-New Haven Hospital for the 10-year-period from 1986 to1995. All cases were further investigated for involvement of endometrial cancers including malignancies without myometrial invasion; histologic subtypes of all malignant tumors were catalogued. Histologic types of endometrial malignancies were characterized according to the WHO classification [12]. All cases were reviewed microscopically and confirmed by a second pathologist. For endometrial cancer with mixed histologic type, the presence of a second component was considered if it involved more than 10% of all available sections containing tumor. The cases of endometrial malignancy involving both EMPs and non-polyp endometrium, were classified into the category of EMPs with cancer involvement.

### Patients groups

For comparative purposes, the patients were divided into 5 age groups: 25–35; 36–45; 46–55; 56–65; and >65 years; and each group was further classified based on an association (or lack thereof) with endometrial carcinoma. A starting point of 25 years of age was selected due to the very low incidence of endometrial polyps in patients below this age. Two patients (ages 18 and 19) were excluded from the study as they did not represent a sufficiently large for statistical analysis group. The proportion of both groups (polyps associated with malignancies (malignant polyps) and polyps not associated with malignancies (benign polyps) were statistically compared for each of the aforementioned age-groups. Subsequently, we merged the younger age groups and preserved the >65 year group, which we referred to as "postmenopausal", since significant differences were observed in this specific subset of patients. Larger age groups were arbitrarily designated as reproductive years (25–45), perimenopause (shortly before or after menopause, 46–65) and postmenopause (>65) and statistically analyzed. The postmenopausal status of all women above the age of 65 was verified and the term "postmenopausal" was occasionally used when referring to this particular age group. Our use of this term, although arbitrary, was important in order to put the emphasis on the fact that any pathomorphological findings in this age group are unlikely to be attributed to changes characteristic of the cycling endometrium.

### Statistical analysis

Chi-square test was used to compare the proportion of malignancy associated EMPs between the age groups and in regards to particular histological type of malignancies.

## Results

**Figure 1:**
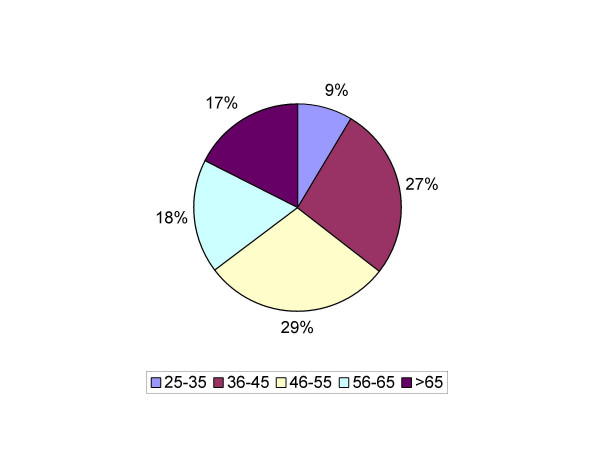
**Frequency of occurrence of benign endometrial polyps by age group**. The frequency of occurrence of EMPs at the time of diagnosis peaked in the age group 46–55 years (29%), followed by 36–45 (27%), 56–65 (18%) and >65 years (17%). The incidence of EMPs in the age group 25–35 years was significantly lower (9%).

**Figure 2:**
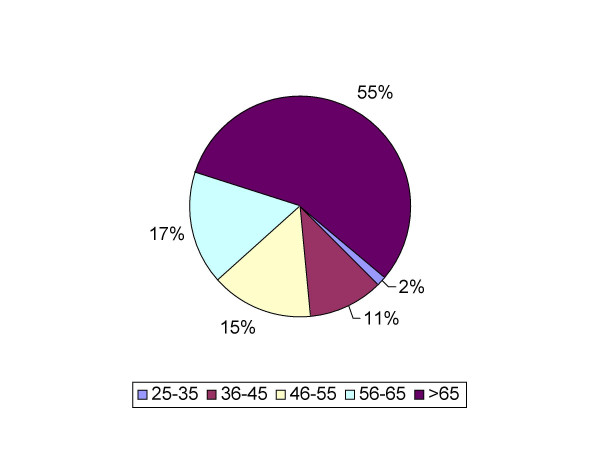
**Frequency of occurrence of malignant endometrial polyps by age group**. In age group 25–35 years, only 2.5% of the EMPs were associated with endometrial malignancy. In contrast, in the age group >65 years, 32% of the EMPs were associated with malignancy.

**Figure 3:**
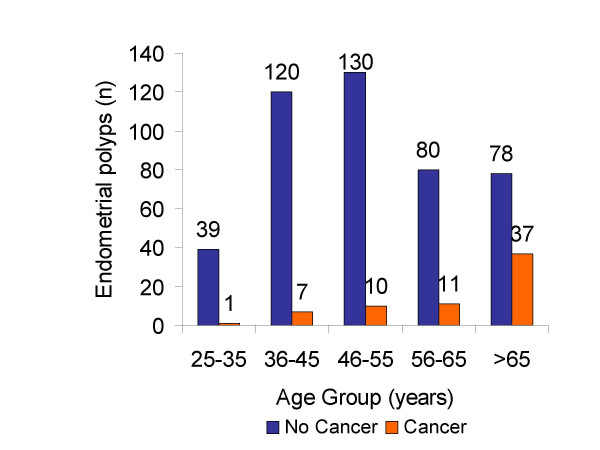
Distribution of benign and malignant endometrial polyps by age group Although the incidence of EMPs at the time of diagnosis in the age group > 65 years was among the lowest, the incidence of malignancy associated EMPs was the highest.

**Figure 4:**
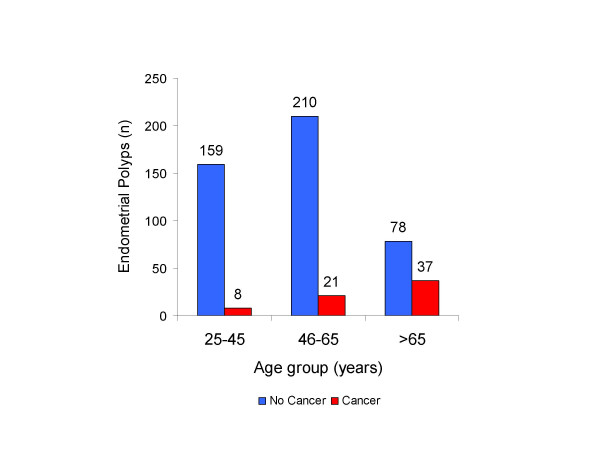
Benign and malignant endometrial polyps by age group

Out of all diagnostic and therapeutic procedures performed over this period, a total of 513 EMP were identified. The latter included 304 (59%) endometrial biopsies/curetting samples, and 209 (41%) hysterectomy specimens. In cases in which endometrial biopsies and hysterectomies both showed presence of EMP, only the hysterectomy specimen was considered. The age of patients ranged from 18–91 years with a median of 54 years. Sixty-six (13%) of 513 EMPs were malignant. The histological subtype distribution of those 66 malignancies included 58 endometrioid (87%), 6 serous (9%), 1 carcinosarcoma, and 1 clear cell carcinoma. No mixed histological type of endometrial cancer was found in our series. The incidence of EMP peaked at age group 46–55 years, which was similar to previous reports. In age group 25–35, only 1 (2.5%) of 40 EMPs was associated with endometrial malignancy. In contrast, 37 (32%) of 115 EMPs were associated with malignancy in the age group >65 years (Figure 1; Figure 2). The frequency of EMPs with malignancy involvement increased with age and reached statistical significance (*p *< 0.001) in the age group >65 years (Figure 3). The most common histological type of malignancy was endometrioid carcinoma, followed by serous carcinoma. The same statistically significant difference for age group >65 years (p < 0.05) remained when larger age groups, including reproductive (25–45), perimenopausal (46–65) and postmenopausal (>65) patients were compared (Figure 4).

## Discussion

Our results indicated a strong age dependent association of endometrial polyps and endometrial carcinoma. A linear relationship in the association rate of EMPs with malignancies and increasing age was observed, with the highest association rate identified in the >65 years age group, where 32% of the EMPs were associated with malignancy. Histological evaluation and characterization of the morphological types of carcinoma demonstrated that the vast majority (87%) of endometrial carcinomas associated with EMPs were of endometrioid, followed by the serous type (9%). These relative proportions of both major histological subtypes are in accordance with the well-known distribution of similar subtypes of endometrial carcinoma in non-polypoid endometrium [12] and thus demonstrates that neither histologic subtype is more likely than the other to develop in a polyp as compared to the adjacent endometrium. The majority of the serous carcinomas developed in the oldest age group (>65 years), whereas the majority of the endometrioid carcinomas occurred in the 46–55 age group, followed by the 36–45 age group. These age distributions are in accordance with the general concept of Type I and Type II endometrial carcinogenesis [13] and provide some evidence suggesting that carcinomas developing in EMPs do not necessarily have clinicopathologic differences from carcinoma arising in the background endometrium. It is well established that serous carcinoma may exist as a minute foci in the endometrium devoid of myometrial invasion and still show extrauterine involvement [14-16]. In a study of EMPs with serous carcinoma involvement with no or minimal invasion, Silva *et al*., [17] reached similar findings: in 6 (37.5%) of 16 cases in that study, there was evidence of extra uterine involvement at presentation. The similarities between the patients who presented with advanced disease and the patients who presented with initial stage disease, suggested that serous carcinoma involving endometrial polyps may represent one aspect of a multicentric disease in which, the entire female genital tract and the abdominal peritoneal surfaces would be at high risk for concurrent or subsequent involvement by serous carcinoma even in the absence of myometrial invasion [17] or the extrauterine disease may represent transtubal metastasis [18,19].

This study also confirms previous findings that EMPs are most prevalent in the perimenopausal age group. The reason(s) for this age-segregation, which has remained remarkably consistent across various studies since the mid-fifties, is unclear. Chavez *et al*., [20] speculated that with the introduction of new minimally invasive technologies (such as office hysteroscopy and sonohysterograms), the demographics of patients with EMPs will change over time as younger women undergoing evaluation for infertility will have "latent" EMPs discovered. However, when the authors compared the mean ages of women with EMPs in 1990 and 1996, there was no statistically significant difference. In addition, multiple EMPs are more prevalent in the postmenopausal women (26%) as compared with their premenopausal (15%) counterparts with EMPs. These findings suggest that the factor, or the constellation of factors responsible for the above mentioned observation is intrinsic to the endometrial polyps and surrounding endometrium condition depending on the age group. Lower incidence of endometrial polyps in the younger age group may be attributed to a possible spontaneous regression mechanism, which is characteristic of the cycling endometrium in young reproductive age women.

Despite the supportive evidence of no difference in the clinico-pathological features and overall distribution of carcinomas arising from EMPs with those arising from non-polypoid endometrium, our data suggest a strong age dependent association between the presence of EMPs and involvement by endometrial carcinoma. The pathogenesis and mechanisms underlying such association are complex and not well established. Recently published data, however, provided some clue of significant differences in receptor expression, response to stimuli, and apoptosis regulation in EMPs compared to benign non-polypoid endometrium which could potentially elucidate some aspects of the possible malignant potential of EMPs. Estrogen and progesterone act as modulators of endometrial proliferation and differentiation through their receptors. Glandular epithelial expression of estrogen and progesterone receptors in polyps is not significantly different from that of the normal cycling endometrium. However, fewer stromal cells express estrogen and progesterone receptors in polyps which suggests that EMPs may result from a decrease in estrogen and progesterone receptors in the stromal cells [21]. In addition, although EMPs depend partially on estrogen receptors and grow in response to estrogen stimulation, their growth is not entirely dependent on them, this is especially so in postmenopausal women. The presence of c-erbB2 over-expression in endometrial polyps, in association with higher proliferation rates were established in a recent study [22]. This finding could explain the presence of polyps showing signs of proliferation even when the adjacent endometrium is atrophic. Thus, C-erbB2 over-expression in endometrial polyps and not in the adjacent atrophic mucosa may render polyps more sensitive to the combination of high gonadotropins and low estrogen levels, which is characteristic in the postmenopausal women.

Another significant histological finding is the glandular epithelia hyperplasia in C-erbB2 -positive polyps as opposed to rather atrophic architecture in C-erbB2 -negative polyps [22]. These findings indicate that the relationship between the expression of estrogen receptors and cell proliferation in normal endometrium and EMPs differ significantly. The balance between mitotic activity and apoptosis, which regulates normal endometrial development in EMPs also shows significant alterations. Bcl-2 is a proto-oncogene, which prolongs the cell survival by inhibiting apoptosis. Bcl-2 expression has been characterized in normal cycling endometrium. Recent studies have also observed that Bcl-2 is strongly expressed in hyperplastic and malignant endometrium [23]. A localized increase in Bcl-2 expression and consequential decline or cessation of apoptosis may be another mechanism underlying the pathogenesis of endometrial polyps [24]. Elevated Bcl-2 expression results in failure of the polyp tissue to undergo normal cycle dependent sequence of proliferation, differentiation and shedding. These data imply that the relationship between receptor expression, cell proliferation and apoptosis in normal and polypoid endometrium differ significantly. Such differences combined with the nonrandom chromosomal aberrations and monoclonality, suggest that EMPs may provide a suitable microenvironment for the development of malignancy, particularly epithelial cancers. In this aspect, the molecular and/or cytogenetic alterations inherent to EMPs in a postmenopausal background could be viewed as factors facilitating and contributing to the process of malignant transformation. Our results showed a strong association of EMPs in postmenopausal patients with endometrial cancer. It raised the possibility that EMPs in postmenopausal women could represent some intermediate stage in the development of carcinoma. A similar suggestion was proposed in a study evaluating the spectrum of pathological findings in Tamoxifen treated breast cancer patients whom develop polyps and carcinoma significantly more frequently than the general population [25]. Also in favor of this hypothesis were the results provided by Silva *et al*., who found that 10 (76%) of 13 Tamoxifen-related endometrial carcinomas were associated with EMPs [26].

One potential limitation of our study is our lack of consideration of the impact of variables such as hypertension, obesity and family history. However, since data regarding such possible confounders were not available to us, we set the goals of our investigation to be primarily focused on age related distribution of coinciding morphologic findings. Although we are aware of the limitations of our study and the introduced analytical bias, drawbacks that certainly pertain to any similar retrospective pathomorphologic study, we feel that we have adequately addressed the proposed investigative tasks according to the initially set scope of the study. By using the database of Yale-New Haven Hospital, we collected and analyzed a significant number of cases over an extensive period of time and thereby our study population constituted an adequate representation of the general population in respect to the morphological parameters we investigated.

In summary, the age distribution, histological subtype distribution, and peak incidence of EMPs was similar to previous reports. In contrast, EMPs in postmenopausal women showed a significantly higher association with malignant tumor involvement. Careful microscopic search for malignancy in patients with multiple risk factors, particularly in postmenopausal women is advised in daily surgical pathology practice.

## Competing interests

The author(s) declare that they have no competing interests.

## Authors' contributions

**DH **wrote the original version of the manuscript.

**OF **made substantial contributions to the content of the manuscript and participated in manuscript preparation.

**MM **collected clinical and pathological data and revised the final version.

**WZ **analyzed and interpreted the data and supervised the entire project.

All authors have read and approved the final manuscript.
